# Prostate cancer survival in sub-Saharan Africa by age, stage at diagnosis, and human development index: a population-based registry study

**DOI:** 10.1007/s10552-021-01453-x

**Published:** 2021-07-10

**Authors:** Tobias P. Seraphin, Walburga Y. Joko-Fru, Shyam S. Manraj, Eric Chokunonga, Nontuthuzelo I. M. Somdyala, Anne Korir, Guy N’Da, Anne Finesse, Henry Wabinga, Mathewos Assefa, Freddy Gnangnon, Rolf Hansen, Nathan G. Buziba, Biying Liu, Eva J. Kantelhardt, Donald M. Parkin

**Affiliations:** 1 Institute of Medical Epidemiology, Biostatistics, Biometrics and Informatics, Martin-Luther-University Halle-Wittenberg, Magdeburgerstrasse 8, 06097 Halle (Saale), Germany; 2 African Cancer Registry Network, INCTR African Registry Programme, Oxford, UK; 3 Nuffield Department of Population Health, University of Oxford, Oxford, UK; 4 Mauritius National Cancer Registry, Candos, Mauritius; 5 Zimbabwe National Cancer Registry, Harare, Zimbabwe; 6 Eastern Cape Cancer Registry, South African Medical Research Council, Tygerberg, South Africa; 7 National Cancer Registry, Kenya Medical Research Institute, Nairobi, Kenya; 8 Registre Des Cancers d’Abidjan, Abidjan, Côte d’Ivoire; 9 Université Mohammed V de Rabat, Rabat, Morocco; 10 Seychelles National Cancer Registry, Victoria, Seychelles; 11 Kampala Cancer Registry, Makerere University School of Medicine, Kampala, Uganda; 12 Addis Ababa City Cancer Registry, Addis Ababa University, Addis Ababa, Ethiopia; 13 Cotonou Cancer Registry, Ministry of Health, Cotonou, Republic of Benin; 14 Namibian National Cancer Registry, Cancer Association of Namibia, Windhoek, Namibia; 15 Eldoret Cancer Registry, Moi University, Eldoret, Kenya; 16 Department of Gynaecology, University Hospital Halle, Martin-Luther-University Halle-Wittenberg, Halle, Germany; 17 International Agency for Research on Cancer, Lyon, France

**Keywords:** Adenocarcinoma of the prostate, Population-based cancer registration, Africa, Survival, Cancer surveillance

## Abstract

**Objectives:**

To estimate observed and relative survival of prostate cancer patients in sub-Saharan Africa (SSA) and to examine the influence of age, stage at diagnosis and the Human Development Index (HDI).

**Patients and methods:**

In this comparative registry study, we selected a random sample of 1752 incident cases of malign prostatic neoplasm from 12 population-based cancer registries from 10 SSA countries, registered between 2005 and 2015. We analyzed the data using Kaplan-Meier and Ederer II methods to obtain outcome estimates and flexible Poisson regression modeling to calculate the excess hazards of death

**Results:**

For the 1406 patients included in the survival analyses, 763 deaths occurred during 3614 person-years of observation. Of patients with known stage, 45.2% had stage IV disease, 31.2% stage III and only 23.6% stage I and II. The 1 and 5-year relative survival for the entire cohort was 78.0% (75.4–80.7) and 60.0% (55.7–64.6), while varying between the registries. Late presentation was associated with increased excess hazards and a 0.1 increase in the HDI was associated with a 20% lower excess hazard of death, while for age at diagnosis no association was found.

**Conclusions:**

We found poor survival of SSA prostatic tumor patients, as well as high proportions of late stage presentation, which are associated with inferior outcome. This calls for investment in health-care systems and action regarding projects to raise awareness among the population to achieve earlier diagnosis and improve survival.

## Introduction

According to GLOBOCAN estimates for the year 2018, prostate cancer was the top cancer in terms of age-standardized incidence rates in males in the majority of countries (118) worldwide and in nearly all of those in sub-Saharan African (SSA) (42) [1]. It is predicted, that just through demographic changes, the annual number of incident prostate cancer cases in Africa will more than double during the next 20 years [2]. In a recent analysis of time trends in prostate cancer incidence in sub-Saharan Africa, we showed that even adjusted for the effect of demographic changes the rates have been increasing annually by 2–10% during the last decade [3]. With a growth rate of 2% throughout the next two decades, the number of cases of prostate cancer will have more than tripled by 2040 [2]. Already today SSA countries are struggling to deal with the burden of cancer. Late presentation of prostate cancer patients has been described in several hospital-based studies while difficulties in access to adequate care of cancer patients in general is a well-known problem of SSA [4–9]. Although prostate cancer is estimated to be the number one cancer in terms of both numbers of cases and deaths in males in most SSA countries [1], there is little information on survival. The few hospital-based studies available have reported wide variations, but mainly poor survival from prostate cancer in SSA [8, 10, 11], yet those estimates have limited generalizability to the general population of the region. Population-based cancer registries originally simply monitored the occurrence of incident cancers, however “the activities of cancer registries have developed far beyond this to include studies of cancer cause and prevention, and to provide the information needed for the planning and evaluation of cancer-control programmes” [12].

Since 2012 the African Cancer Registry Network (AFCRN) has been the partner of the International Agency for Research on Cancer (IARC), facilitating population-based cancer registration in SSA as a regional hub of the Global Initiative for Cancer Registration (GICR) [13]. Data on survival from prostate cancer have been published from individual registries [14–17]. However, a broad and in depth analysis of the population-based survival of prostate cancer patients in SSA and an analysis of influencing factors is not available.

In this comparative registry study, we estimate 1-, 3- and 5-year observed and relative survival for 12 population-based cancer registries from 10 SSA countries and examine the influence of age, stage at diagnosis, and the Human Development Index [18].

## Patients and methods

### Study population

We obtained data from 12 population-based cancer registries from 10 SSA countries, all members of the African Cancer Registry Network (AFCRN, https://afcrn.org/): Cotonou (Benin), Abidjan (Côte d’Ivoire), Addis Ababa (Ethiopia), Eldoret (Kenya), Nairobi (Kenya), Mauritius, Namibia, Eastern Cape (South Africa), Seychelles, Kampala (Uganda), Bulawayo (Zimbabwe) and Harare (Zimbabwe). In 2016 we invited those registries that were members of AFCRN that were capable of providing follow-up data for a minimum of 3 years, and ideally 5 and the aforementioned agreed to participate. From each individual registry, we took a simple random sample from lists of incident prostate cancer cases (ICD-O-10: C61) in the AFCRN database, registered between 2005 and 2015. For Harare (Zimbabwe) we took one random sample of cases among black men and one of white men from the same period. Since active follow-up is resource intensive in this setting, the sample size for each registry was determined by the feasibility of obtaining follow-up information. If passive follow-up was used, a larger number of patients could be included.

Primary prostate cancer cases of at least 15 years of age were eligible for sampling. Recurrences and cases registered on the basis of a death certificate only (DCO) were excluded. We measured the follow-up time from the date of incidence to the date of last contact alive, to the date of death or to the closing date of the study for the corresponding registry, whichever occurred first.

Cases were excluded from survival analyses due to the following criteria: (1) Less than one day of follow-up time; (2) incoherent dates (i.e. the registered date of incidence lies after the date of last contact); (3) double registrations; (4) found not to be prostate cancer during the follow-up process; (5) initially diagnosed before the study period of the registry (registered relapses); (6) unknown age.

### Covariates

#### Vital status

We investigated vital status using means of active and passive follow-up. All registries, apart from Mauritius used active follow-up methods. In Mauritius, the follow-up was done passively, by linking the records to the death registry. In 2012, the completeness of this death registry was estimated to be 100% [19]. For verification, the registry performed active follow-up of 10% of the presumably living patients and found all of this 10% sample to be still alive on 31st December 2013. Accordingly, we assumed patients to be still alive, if they were not registered in the death registry.

In all other registries, active follow-up was performed, using medical records to determine the patient’s vital status and date of last contact. For patients not known to have died, the registry staff augmented this information, if possible, with phone calls and sometimes home visits to the patients and their relatives. We censored patients “alive” at the date of last contact, if vital status (alive or dead) was unknown at the closing date (Appendix Fig. 3).

#### Stage at diagnosis

At the time of registration, the registry staff abstracted information on clinical stage at diagnosis. For most registries this included tumor-node metastasis (TNM) assessment. For some registries additional information was available on prostate specific antigen (PSA) levels at time of diagnosis and/or the Gleason Score. We used the AJCC Cancer Staging Manual 8th edition, of the American Joint Cancer Committee (AJCC) [20] to classify each prostate cancer case to one of the four stage groups (I–IV). Since for some patients only PSA level and/or Gleason score was available, a stage was assigned on the basis of this information alone, assuming the other risk factors to be at minimum level. Accordingly, we grouped all prostate cancer cases in one of the following groups: “Stage I–II”, “Stage III, “Stage IV” and “Stage unknown”. For the registries of Mauritius and Eastern Cape (South Africa) no stage information was available.

#### Basis of diagnosis

The registries code the most valid basis of diagnosis [21] they can find for each cancer patient. We grouped “Morphologically verified” cases as those with histopathological verification of the primary tumor (the majority), and a few cases with cytological diagnosis or histopathological verification of metastases.

#### Human development index

According to the United Nations Development Programme (UNDP), the Human Development Index (HDI) is a “composite index measuring average achievement in three basic dimensions of human development—a long and healthy life, knowledge and a decent standard of living” [22]. The HDI “is perhaps the most popular index used to assess countries’ well-being levels across the globe” [18]. For those registries covering sub-national populations, we used the more precise Sub-national Human Development Index (SHDI) (https://globaldatalab.org/) [18] to allow for the wide differences of well-being within countries in SSA. For Namibia, where registry coverage is not complete at the national level, we estimated a weighted average HDI, based on the SHDI of the 13 regions of the country and the number of cases from each in the random sample. In order to compare between the registries, the HDI value of 2013 was chosen.

### Statistical analyses

#### Observed survival

Following exclusion of ineligible cases (as described above), we estimated observed survival (OS) probabilities at 1, 3 and 5 years of follow-up, applying the semi-complete [23] approach. We plotted Kaplan–Meier (KM) curves of observed survival probabilities, as well as observed survival stratified by HDI group, age and stage group at diagnosis.

We used R, Version 3.6.3 [24] in the integrated development environment RStudio, Version 1.2.5033 [25] with the packages “survival” [26] and “survminer” [27].

The percentage of cases with morphological diagnosis (MV%) was calculated as an indicator of data quality [28]. We estimated the median follow-up time for all cases, including those with a known event of death.

#### Relative survival

To adjust for mortality due to causes of death other than prostate cancer, we calculated crude and age-standardized Ederer II relative survival (RS) at 1, 3 and 5 years of follow-up, using the “relsurv” package [29] for R. We obtained the national life tables as five-year age-specific death rates by calendar year, sex and country from the WHO Mortality database [30] and expanded them using a Poisson regression model implemented in the “rcsgen” [31] command for STATA 15, to obtain complete life tables by one year age group (more information in the Supplement). We performed direct age-standardization by applying the age-specific weights of the International Cancer Survival Standard-1 for prostate cancer [32], but, since the numbers of subjects in the upper and lower age groups of the standard were very small, when stratifying by registry, we used just three broad age groups: 15–64, 65–74, 75–99.

#### Estimation of average survival

We estimated average 5-year survival for the ten countries under observation, adjusting for the different size of the datasets from each country, using the method of Abdel-Rahman et al. [33]. In brief: we weighted the mean of the 5-year survival from each country by the number of prostate cancer patients included as a proportion of the total cases for that country, as estimated by GLOBOCAN 2018 [1]. This does not necessarily imply that regional survival estimates can be extrapolated to the national level.

#### Assessing loss to follow-up

We assessed the proportions of patients lost to follow-up (LFU) at 1, 3 and 5 years. Since these proportions were above 10%, and in such cases it is desirable to investigate if censoring is at random, we performed an “inverse” Cox proportional hazards model with LFU as the outcome and adjusted for age and stage at diagnosis for year 1 and year 5.

#### Assessing the potential of 5-year follow-up

For all registries (except Mauritius) the closing date for follow up was 31st December, 2017, so that we calculated the potential follow up period for each patient as the difference between the date of incidence and the closing date. If this period was greater than 5 years, we considered this patient to have a potential of 5-year follow-up.

#### Modeling excess hazards

We used univariable and multivariable Poisson regression models adjusted for stage group, HDI as a continuous variable and age group at diagnosis, splitting time into monthly intervals and using restricted cubic splines, to model excess hazards of death in RS framework for prostate cancer patients [34].

## Results

Mauritius, Namibia and Seychelles had national population coverage, the registry in Eastern Cape (South Africa) covers a rural area and all other registries cover urban areas. From these 12 population-based cancer registries a total 1752 cases were randomly selected, representing a 44% of the total prostate cancer cases (after exclusion of death certificate only cases) registered within the study period (Table 1).

**Table 1:** Total number of prostate cancer cases registered, included and excluded, data quality indicator by population-based cancer registry

Country	Registry	HDI in 2013^1^	Period of diagnosis	Total of prostate cancer patients during study period	No. excluded due to DCO (%)	Random sample, (sampling fraction %)	Included for survival analyses, (fraction of random sample, %)	MV, %
Benin	Cotonou	0.580	2013–2014	54	0 (0)	54 (100)	43 (80)	53
Côte d’Ivoire	Abidjan	0.548	2013–2014	286	0 (0)	160 (56)	127 (79)	65
Ethiopia	Addis Ababa	0.653	2012	49	0 (0)	49 (100)	45 (92)	73
Kenya	Eldoret	0.546	2009–2013	177	7 (4)	75 (44)	23 (31)	74
	Nairobi	0.622	2009–2013	866	47 (5)	149 (18)	134 (90)	75
Mauritius	Mauritius	0.775	2005–2009	340	9 (3)	331 (100)	326 (99)	96
Namibia	Namibia	0.665^2^	2012–2013	443	0 (0)	80 (18)	35 (44)	74
Seychelles	Seychelles	0.782	2008–2013	140	10 (7)	130 (100)	119 (92)	95
South Africa	Eastern Cape	0.644	2008–2013	260	0 (0)	260 (100)	201 (77)	49
Uganda	Kampala	0.621	2009–2013	559	5 (1)	150 (27)	114 (76)	42
Zimbabwe	Bulawayo	0.623	2012–2013	135	21 (16)	60 (53)	50 (83)	54
	Harare (black)	0.599	2009–2013	905	168 (19)	200 (27)	148 (74)	91
	Harare (white)	0.599	2009–2013	66	12 (18)	54 (100)	41 (76)	93
Total			2005–2014	4280	279 (7)	1752 (44)	1406 (80)	75

*DCO* death certificate only, *MV* morphologically verified

^1^Human Development Index (http://hdr.undp.org/en/data and https://globaldatalab.org/), *Levels* Very High HDI (0.800–1.000), High HDI (0.700–0.799), Medium HDI (0.550–0.699), Low HDI (0.000–0.549)

^2^National weighted average (by No. of cases per subregion) of the subnational HDIs (https://globaldatalab.org/)

Table 1 shows, for each registry, the total number of prostate cancer patients from the catchment area during study period, the number (and %) of DCO cases (not eligible for the study sample), and the number of cases in the random sample (and sampling fraction). Also shown is the number (and percentage) of the cases in the random sample included for survival analysis, following exclusion on non-eligible cases, as described above, their mean age and the percentage of morphologically verified (MV) cases.

The sampling fraction ranged from 18% in Namibia, to 100% in six registries. The proportion of MV cases ranged from 42% in Kampala (Uganda) to 96% in Mauritius. Following exclusions, 1406 prostate cancer patients were included in the survival analysis, representing 80% of our random sample. During a total of 3613 person-years of observation, there were 763 deaths, and the individual median time of follow-up was 1.78 years (Table 2), without excluding the deaths from the calculation. The HDI ranged from 0.546 in Eldoret (Kenya) to 0.782 in Seychelles.

**Table 2:** Patient characteristics: mean age at diagnosis, median years of follow-up and observed (all-cause) survival and loss to follow-up

Country	Registry	Mean age at diagnosis (SD), years	No. of cases included	Year 1			Year 2 and 3			Year 4 and 5		Median follow-up time (IQR), years
				No of deaths (%)^2^	LFU (%)^2^	Observed 1-year survival % (95% CI)	No of deaths (%)^2^	LFU (%)^2^	Observed 3-year survival % (95% CI)	No of deaths (%)^2^	Observed 5-year survival % (95% CI)	
Benin	Cotonou^1^	69.5 (9.8)	43	8 (19)	21 (49)	72 (57–91)	9 (64)	0 (0)	26 (12–54)	–	–	0.56 (1.68)
Côte d'Ivoire	Abidjan	68.0 (9.8)	127	20 (16)	46 (36)	78 (70–87)	30 (49)	5 (8)	37 (28–50)	5 (19)	30 (21–43)	0.79 (2.43)
Ethiopia	Addis Ababa^1^	67.7 (9.9)	45	17 (38)	3 (7)	60 (47–77)	11 (44)	7 (28)	29 (17–49)	–	–	1.11 (2.08)
Kenya	Eldoret	74.2 (9.8)	23	2 (9)	6 (26)	89 (75–100)	7 (47)	0 (0)	47 (29–78)	2 (25)	36 (19–67)	1.37 (3.14)
	Nairobi	67.4 (10.0)	134	18 (13)	41 (31)	83 (76–90)	11 (15)	14 (19)	69 (60–79)	7 (14)	58 (48–70)	1.52 (4.34)
Mauritius	Mauritius	71.5 (9.7)	331	73 (22)	0 (0)	78 (74–83)	73 (29)	0 (0)	56 (50–61)	30 (17)	46 (41–52)	4.08 (3.83)
Namibia	Namibia	66.5 (9.1)	35	5 (14)	3 (9)	85 (73–98)	4 (15)	6 (22)	70 (56–89)	1 (6)	66 (51–86)	2.84 (3.88)
Seychelles	Seychelles	70.8 (8.4)	119	21 (18)	0 (0)	82 (76–89)	29 (30)	3 (3)	58 (49–67)	17 (26)	41 (33–52)	3.34 (3.49)
South Africa	Eastern Cape	72.0 (10.1)	201	73 (36)	29 (14)	60 (54–68)	32 (32)	23 (23)	37 (30–46)	10 (23)	28 (21–37)	1.00 (2.64)
Uganda	Kampala	69.5 (9.0)	115	34 (30)	18 (16)	66 (57–76)	19 (31)	8 (13)	44 (35–56)	5 (14)	38 (29–49)	1.25 (3.78)
Zimbabwe	Bulawayo^1^	74.4 (8.1)	50	24 (48)	14 (28)	37 (24–56)	7 (58)	4 (33)	11 (4–34)	–	–	0.15 (0.91)
	Harare (black)	71.4 (9.7)	149	50 (34)	3 (2)	66 (59–74)	26 (27)	1 (1)	47 (40–56)	14 (21)	36 (29–45)	2.54 (4.25)
	Harare (white)	73.1 (8.3)	41	11 (27)	2 (5)	72 (59–87)	4 (14)	3 (11)	61 (47–79)	4 (19)	49 (35–69)	3.34 (4.06)
Total	Total	70.5 (9.7)	1406	355 (25)	186 (13)	72.1 (69.6–74.6)	262 (30)	74 (9)	49.2 (46.4–52.1)	96 (18)	39.1 (36.3–42.2)	1.78 (4.03)

*CI* confidence interval, *IQR* interquartile range, *LFU* loss to follow-up, *SD* standard deviation

^1^Registries without a potential of 5-year follow-up

^2^Percentages refer to the number at risk at the beginning of the time intervals of observation

The mean (SD) age at diagnosis was 70.5 (9.7) years, and ranged from 66.5 (9.1) years in Namibia, to 74.2 (9.8) in Eldoret (Kenya) (Table 2). Distribution of age by registry can be seen in Appendix Fig. 4. Age distribution of our cohort was compared with that of all prostate cancer cases in the target populations during the years concerned, and found to be representative. Information on stage was only available for 40.5% of patients from the 10 registries contributing staging information (i.e. excluding Mauritius and Eastern Cape, South Africa). Of patients with a known stage, 45.2% had stage IV disease, 31.2% stage III and only 23.6% stage I and II. The proportion of “Stage unknown” varied widely between the registries and ranged from 17% in Namibia, to 76% and 75% in the cohort of white and black men in Harare (Zimbabwe), respectively. The highest proportion of Stage I and Stage II disease was found in Namibia, Seychelles and Nairobi (Kenya), with 31%, 21% and 13%, respectively (Appendix Fig. 5).

### Assessing Loss to follow-up

LFU was the highest during the first year; for the entire cohort it was 13%. The proportion of LFU in the first year ranged from 0 and 2% in Seychelles and Harare (Zimbabwe) blacks, to 49 and 36% in Cotonou (Benin) and Abidjan (Côte d’Ivoire). Our Cox model, adjusted for stage and age group, showed that censoring was at random at year one, as well as during the whole study period.

The registry cohorts from Cotonou (Benin) and Bulawayo (Zimbabwe) had no potential for a 5-year follow-up. Since only three patients from Addis Ababa had a potential of 5-year follow-up, we did not estimate 5-year survival for this registry. Nairobi (Kenya) had the lowest percentage of cases with a complete 5-year follow-up (51%), whereas Mauritius and blacks from Harare (Zimbabwe) had the highest with 100% and 96%, respectively (Appendix Table 4).

### Survival statistics for all ages by registry

**Fig. 1:**
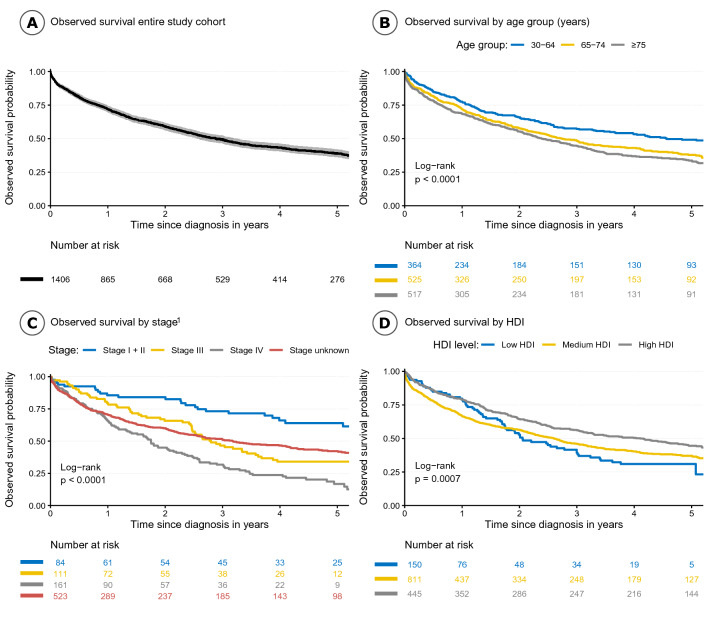
Observed (all-cause) survival for the entire study cohort (**A**), by age group (**B**), by stage (**C**), and Human Development Index (HDI) (**D**), Source HDI (http://hdr.undp.org/en/data and http://globaldatalab.org/). ^1^Mauritius and Eastern Cape (South Africa) excluded, since no staging information was available

For the whole study cohort, the observed Kaplan–Meier survival probability (95% CI) for prostate cancer patients was 72.1% (69.6–74.6) at year one, 49.2% (46.4–52.1) at year 3 and 39.1% (36.3–42.2) at year 5 (Fig. 1A, Table 2). The youngest age group had the highest observed survival (Fig. 1B). The 5-year observed survival probability was highest in Namibia and lowest in Eastern Cape (South Africa) (Table 2, Appendix Fig. 6).

The 1-, 3- and 5-year relative survival for the entire cohort was 78.0% (75.4–80.7), 62.9% (59.4–66.7) and 60.0% (55.7–64.6) (Appendix Fig. 7). The values varied by registry, with the highest values of 5-year relative survival found in Namibia, Nairobi (Kenya), in whites of Harare (Zimbabwe) and in Eldoret (Kenya). The lowest values of 5-year relative survival were found in Eastern Cape (South Africa) and in Kampala (Uganda).

**Fig. 2:**
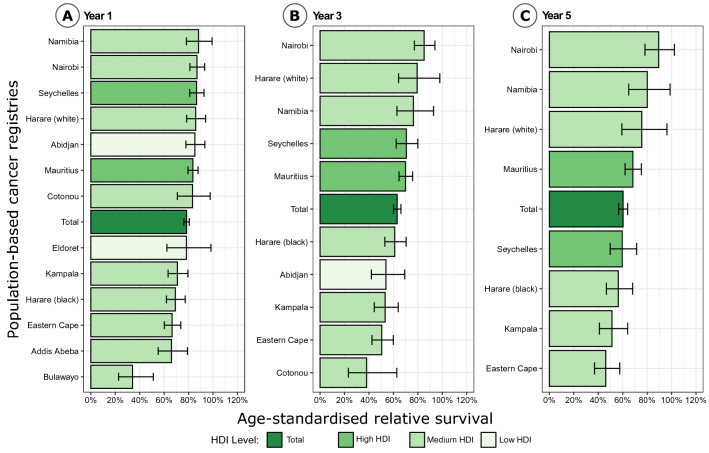
Comparison of 1- (**A**), 3- (**B**) and 5-year (**C**) age-standardized relative survival with 95% confidence intervals (CI) by registry and Human Development Index (HDI)

Figure 2 shows the 1-, 3- and 5-year age-standardized relative survival (ASRS) in the different registries. At year 5 we found the highest values for Nairobi (Kenya) and white patients in Harare (Zimbabwe) and the lowest values for Eastern Cape (South Africa). The ASRS also varied within countries. E.g. in Zimbabwe at year 1, where the cohorts from the capital Harare had a better outcome than the cohort from Bulawayo. The ASRS also varied between the white and the black patients from Harare (Zimbabwe), with the whites having one of the best ASRS after 5 years and the blacks having one of the poorest. The ten countries under observation had an estimated average relative survival (taking into account the different sample sizes from each country) of 73.1% (62.5–85.9) at year 1, 49.7% (36.9–69.0) at year 3 and 55.3 (42.1–73.2) at year 5. However, this sample was mainly from urban populations and is not representative for the whole of SSA.

### Survival by age at diagnosis and registry

The oldest age group (> = 75) had a significantly better relative survival probability than both younger groups (< 65, 65–74) with 5-year RS point estimates (95% CI) of 74.9% (65.3–85.9), 56.1%(49.8–63.2) and 51.8% (45.7–58.7), respectively (Appendix Table 5). For most registries we observed the highest relative survival point estimates in the oldest age group at all three evaluated time points. This was not the case for Addis Ababa (Ethiopia) and Namibia, where the highest values were found in the younger age groups.

### Survival by stage at diagnosis

We observed differing KM survival by stage at diagnosis for the entire cohort (Mauritius and Eastern Cape (South Africa) excluded). At five years, those with Stage I + II disease (64.0% [53.1–77.0]) had significantly higher point estimates than those with Stage III (34.1% [25.3–45.9]) and Stage IV disease (16.8% [10.7–26.3]) (Fig. 1C). This pattern was also observed in relative survival estimates for the entire cohort and within registries. The relative survival in each stage group, varied between the registries yet the confidence intervals were mainly wide and overlapping (Appendix Table 6).

### Excess hazard ratio

Stage III and Stage IV at diagnosis were associated with a three- and sevenfold risk of death compared to Stage I + II at diagnosis (Table 3). When adjusting for age at diagnosis and HDI, we observed a similar independent association. An increase of the HDI by one decimal point (0.1) decreased the risk of death by 20% (95% CI: 9–30%) in our model, adjusted for age and stage at diagnosis. Age at diagnosis was not associated with the hazard of death in either the univariable or in the adjusted model. We did not find any evidence in our models for an interaction between age and stage at diagnosis.

**Table 3:** Prostate cancer excess mortality hazard by age and stage at diagnosis and HDI

	No. of cases	Univariable analysis		Multivariable model^1^	
		Excess hazard ratio (95% CI)	*p* Value	Excess hazard ratio (95% CI)	*p* Value
Age at diagnosis (years)					
< 65	364	Reference		Reference	
65–74	525	1.17 (0.91–1.51)	0.213	1.19 (0.93–1.53)	0.173
75 +	517	0.85 (0.63–1.14)	0.281	0.92 (0.68–1.24)	0.584
Stage at diagnosis					
Stage I + II	84	Reference		Reference	
Stage III	111	3.18 (1.12–9.04)	0.030	2.83 (1.04–7.68)	0.042
Stage IV	161	6.93 (2.61–18.38)	< 0.001	6.16 (2.43–15.61)	< 0.001
Stage unknown	1050	3.70 (1.42–9.61)	0.007	3.51 (1.42–8.71)	0.007
HDI^2^ (unit = 0.1)	1406	0.78 (0.68–0.89)	< 0.001	0.80 (0.70–0.91)	0.001

*CI* Confidence interval, *HDI* Human Development Index

^1^Adjusted for age at diagnosis, stage at diagnosis and sub-national HDI

^2^Human Development Index (http://hdr.undp.org/en/data and https://globaldatalab.org/)

## Discussion

This comparative analysis—to our knowledge, the first of its kind from sub-Saharan Africa—evaluates the survival of prostate cancer patients from 10 different countries, incorporating data from 12 population-based cancer registries and assesses the influence of age, stage at diagnosis and Human Development Index. We used random sampling for inclusion of cases, although the size of the sampling fractions and accordingly the confidence intervals for our estimates varied between registries. The total sample of 1752 men included 44% of all patients registered with prostate cancer in the participating registries during the study period.

The survival estimates varied widely between registries and countries, as well as within countries and for Harare (Zimbabwe), between the racial groups. We found a 1-, 3- and 5-year observed (all-cause) survival (95% CI) for all 13 cohorts of prostate cancer patients of 72.1% (69.6–74.6), 49.2% (46.4–52.1) and 39.1% (36.3–42.2), respectively, while the ASRS was at 78.4% (76.2–80.6), 63.1% (60.1–66.1) and 60.3% (56.7–64.1), respectively. The ten countries under observation had an estimated average relative survival (taking into account the different sample sizes from each country) of 55.3 (42.1–73.2) at year 5. Nearly half of the patients with staging information had Stage IV disease. In flexible Poisson regression analysis, we found late stages of prostate cancer associated with increased excess hazards, compared to early stages and a 0.1 increase in the HDI to be associated with a 20% lower excess hazard of death. We did not find an association between age at diagnosis and the hazard of death in prostate cancer patients. It is possible that the lack of an association between hazard of death and age is due to confounding by stage; although this was adjusted for in the model, the adjustment would be far from complete, given the high proportion of cases with missing stage data.

The poor observed survival is to be expected given the advanced stage and age of prostate cancer patients (mean age 70.5 years in our study). Relative survival provides an estimate of the probability of surviving prostate cancer (excluding death from other causes), while comparisons between different series requires adjustment for age (if survival is related to age). Comparing our results of the ASRS to high income countries, like the US, Germany or the UK, where the 5-year age-standardized net survival in 2010–2014 was estimated to be at 97.4, 91.6 and 88.7% [14], respectively, we revealed that the average outcome of prostate cancer patients in SSA is rather poor. However, survival from prostate cancer in high income countries was much lower only a few decades ago. For example, in the US, the 5-year relative survival increased from 70% in the period of 1975–1979, to 99.3% in 1995–2000[35]. In the registry of Kampala (Uganda) the 5-year ASRS for prostate cancer patients was reported to be 46.9% during 1993–1997[16], while in our study it was at 51.2%. Data from Harare (Zimbabwe) from the same period showed a 3-year RS for black men of 27.1% and a 5-year survival for white men of 83.7% [17], respectively. In our study those estimates are at 59.9 and 76.8%, respectively. The survival of cancer patients is a product of a multitude of factors and it is therefore not easy to determine any single reason for the low survival of SSA prostate cancer patients, and for the variations we observe between and within countries. In high income countries, the implementation of routine and opportunistic screening for prostate cancer in asymptomatic men by prostate specific antigen (PSA) testing has been a major factor causing the very high survival currently observed, with much of the longer survival times being a consequence of the so called lead-time bias introduced through over-diagnosis of indolent cancers [36]. In SSA there are no systematic screening programs in place, and there are no data on the prevalence of opportunistic PSA testing. A few studies indicate that PSA screening uptake is sparse in SSA [37, 38]. The rising incidence rates all over SSA in a recent trend analysis of population-based cancer registry data is believed to be linked to rising usage of PSA testing [3].

Another factor influencing the survival of prostate cancer patients is the stage at diagnosis. The majority of patients in our cohort were diagnosed at Stage III and Stage IV. This is in line with most (mainly hospital-based) studies from SSA [9, 39, 40]. The proportion of metastatic disease in high income countries is much smaller (6% in the USA for example [41]), than in our study, where nearly one in two patients (with known stage) was metastatic. As expected, late stage III and stage IV disease were associated with a higher excess hazard of death, even when adjusting for age and HDI.

It is likely that the high proportion of late stage disease is due to lack of awareness of the disease. Only 54% of 545 men in a cross-sectional study from Kampala ever heard about prostate cancer [42]. A recent review from Baratedi et al., similarly pointed towards lack of knowledge and a multitude of misconceptions about the disease. This study also identified lower education and socioeconomic status as barriers to prostate cancer screening on the patient level [43]. These factors are also known to influence the outcome of prostate cancer patients in general [44]. Since we had no information on individual socioeconomic status, we adjust for this on registry level using the HDI as covariate, which comprises information on life expectancy, education level and gross per capita income [18, 22]. We found a higher HDI to be associated with a reduction of the excess hazard of death. Since we modeled in a relative survival setting, which already adjusts for the influence of the background population’s life expectancy, this association will be mainly driven by the influence of education level and gross per capita income. Regions with a higher gross domestic product are likely to have better health-care systems with better access to early detection and adequate treatment, as well as to post-treatment follow-up. A retrospective hospital-based study from South Africa found that patients wait an average of three months to receive the results of their prostate biopsy and to have their treatment planned [45]. It is estimated that 93% of the population of SSA have no access to timely, safe and affordable surgery and anesthesia [5]. A reason among others is likely to be that the region has the least surgical workforce worldwide [46].Shortcomings in the access to radiotherapy, as well as problems with the few functioning radiotherapy machines are a well-known problem in low income countries and especially in this region [4, 6, 47]. Yet a study from a tertiary referral center in Ghana showed that even in this setting the provision of adequate radiotherapy is possible and reports high 5-year observed survival rates (96%) of non-metastatic patients in their clientele [10].

It is possible also that there is a genetic component to the poor survival of men with prostate cancer in SSA. Men of African ancestry not only have been associated with a higher risk of developing prostate cancer, but also with more aggressive disease [48]. Nevertheless, the socioeconomic factors confounding this association are likely to be the principal reason for the racial survival disparities [49–51].

The differences we saw in 5-year relative survival between age groups, were not observed in our model—even in the multivariable (adjusted) analysis. Possibly the pattern observed in the pooled relative survival analysis is artificially influenced by the large proportion of patients in the oldest age group coming from Mauritius (67 of 181 patients at risk in years 4 and 5). Another reason is that relative survival estimation makes use of national lifetables, in which the mortality rates are too pessimistic for the background mortality of men with prostate cancer in the populations served by the registries. Most are in relatively affluent regions of their countries—the capital cities—which will artificially inflate the estimates of relative survival of our patients. Regionally stratified lifetables would reduce such bias, but are not readily available at the moment.

Stage was unknown for around 60% of patients from registries contributing stage information. Cancer registrars can only abstract staging information, if they are sufficiently trained, have access to medical records and if, after all, cancer stage had been assessed by physicians and was documented in the record. This problem is being addressed by the development of simplified staging protocols, which can be used by cancer registrars to allocate stage at diagnosis, in the absence of documented stage in the case record [52].

We used the HDI as a registry-level substitute covariate for unavailable patient-level socioeconomic data. Allocating socioeconomic status based on residential-level indices is now a very widely used technique, although it incorporates misclassification at the individual level [53], and, in our study, is also completely confounded with the actual cancer registry.

In order to minimize any potential bias due to incompleteness of registration, we only included AFCRN registries, which are evaluated as registering at least 70% of their target population [13]. Five of our registries (Eastern Cape, Harare, Kampala, Nairobi, and Seychelles) were included in Cancer Incidence in Five Continents during the relevant period [54]. Several studies have investigated aspects of registration practice to ascertain whether they can explain observed survival differences between countries, finding that particular registration differences are unlikely to impact greatly on survival differences [55]. A large number of patients were lost to follow up (LFU), especially during the first year of follow-up. The Cox-models suggest that LFU at year 1 and during the whole period, was not associated with age or stage and thus was considered to be random.

We analyzed data from 12 population-based cancer registries from 10 SSA countries, giving insight into the survival experience of prostate cancer patients in the general population. We show that survival of prostate cancer patients in SSA is generally poor, but differs widely between and also within different countries, while late stage disease and a lower Human Development index were associated with a substantially increased risk of death. More studies are needed to evaluate and adjust for the influence of patient-level socioeconomic factors, treatment and comorbidity. However, we believe that raising awareness of the disease in the general population to mitigate late stage presentation, as well as investments in training and equipment of health-care systems to improve the patterns of care would lead to a reduction of unnecessary early deaths from a disease that has rather good prognosis in more affluent regions of the world.

## Figures and Tables

**Fig. 3:**
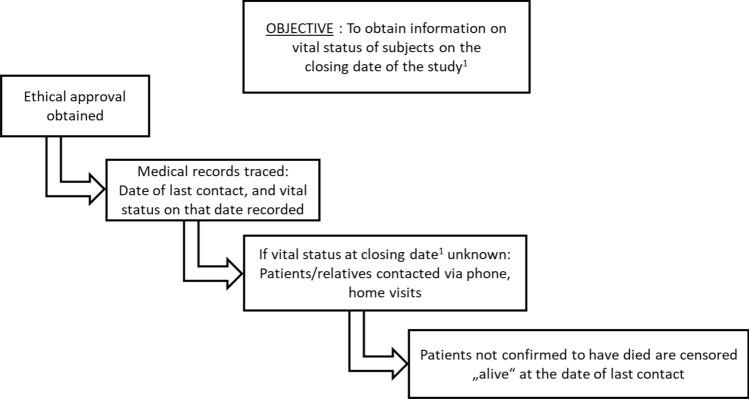
Process of patient follow-up.^1^31st December, 2017, (for Mauritius: 31st December, 2013)

**Fig. 4:**
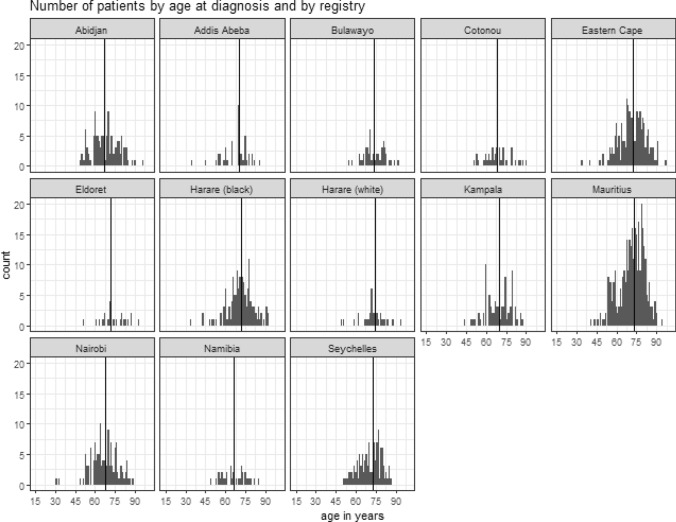
Number of patients by age at diagnosis in years, by registry; black vertical lines indicate median age per registry

**Fig. 5:**
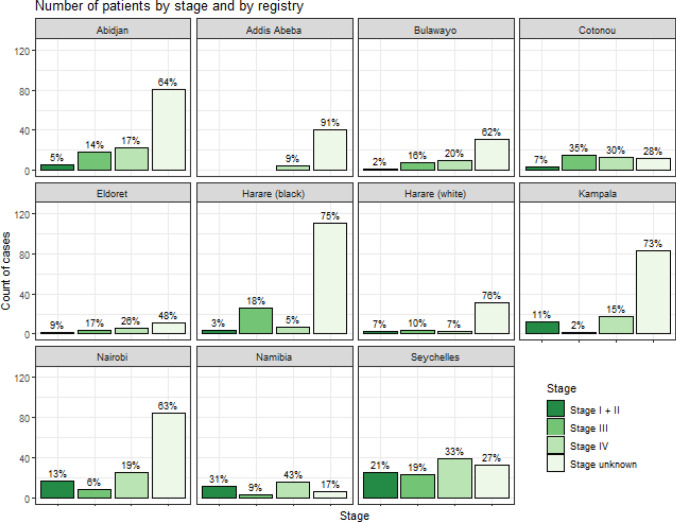
Distribution of stage by registry (Mauritius and Eastern Cape (South Africa) excluded)

**Fig. 6:**
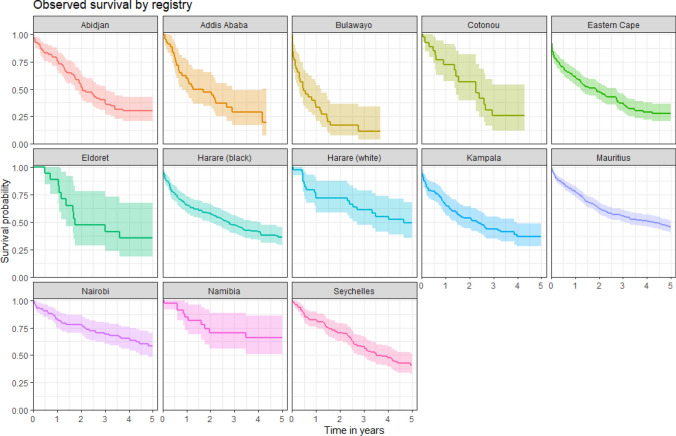
Kaplan–Meier overall survival probabilities (95% confidence intervals) by registry

**Fig. 7:**
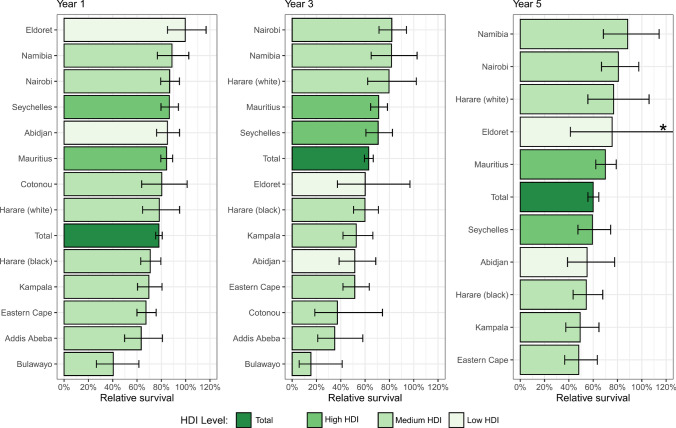
1-, 3- and 5-year relative survival with 95% confidence intervals (CI) by registry and Human Development Index (HDI); *The upper limit of Eldoret’s 95% CI is at 164%

## Data Availability

The data that support the findings of this study are available from the corresponding author upon reasonable request, which will be evaluated by the AFCRN research committee. Details of the data application process are outlined on the AFRCN website (http:/afcrn.org/index.php/research/how-to-apply/76-research-collaborations).

## Appendix methods

### Modeling of lifetables

Single year and 5-year-age abridged lifetables for the years 2000–2016 at national level was retrieved from the WHO Global Health Observatory. We obtained age-specific death rates, calculated from information on deaths among persons in the age group at age x during a given time period and the total person-years for the population in the same time period. A full description of the methods is available elsewhere: https://www.who.int/healthinfo/statistics/LT_method.pdf?ua=1.

The number of deaths and person-time by sex, year and country were used to estimate mortality rates using a Poisson regression and a flexible function to expand the abridged age groups (0–4, 5–9, 10–14 … 80 +) to single ages (0, 1, 2, 3, 4, 5 … 99). Briefly, we used the number of deaths and person-time by year and sex for each country separately. Smoothed age-specific mortality rates were derived using Poisson regression modeling by piecewise and spline function using eight knots with locations at ages 0–10 (three knots), 15–30 (three knots) and 50–85 + (two knots). The method chosen was fully described and explored by Rachet and colleagues (2015): https://bmcpublichealth.biomedcentral.com/track/pdf/10.1186/s12889-015-2534-3 (Figs. 3, 4, 5, 6, 7; Tables 4, 5, 6).

**Table 4:** Registries with potential for 5-year follow-up time

Country	Registry	Period of diagnosis	No. of cases included for survival analyses	No. of cases with potential of 5-year FU	No. of cases with complete (alive or dead) 5-year FU (%)	
					Alive	Dead
Côte d'Ivoire	Abidjan	2013–2014	127	47	1 (2)	25 (53)
Ethiopia	Addis Ababa	2012	45	3*	0 (0)	2 (67)
Kenya	Eldoret	2009–2013	23	17	4 (24)	7 (41)
	Nairobi	2009–2013	134	103	28 (27)	25 (24)
Mauritius	Mauritius	2005–2009	331	255	115 (45)	140 (55)
Namibia	Namibia	2012–2013	35	20	9 (45)	3 (15)
Seychelles	Seychelles	2008–2013	119	92	29 (32)	54 (59)
South Africa	Eastern Cape	2008–2013	201	113	17 (15)	77 (68)
Uganda	Kampala	2009–2013	115	103	23 (22)	51 (50)
Zimbabwe	Harare (black)	2009–2013	149	94	34 (36)	56 (60)
	Harare (white)	2009–2013	41	41	16 (39)	19 (46)
Total			1320	888	276 (31)	459 (52)

*Since there were only three cases, we did not assess 5-year survival for Addis Ababa (Ethiopia)

**Table 5:** Age-specific and age-standardized relative 1-, 3- and 5-year survival by registry

Registry	Year 1 RS				Year 1 ASRS
	< 65	65–74	> = 75	All ages	
Abidjan	83.8 (70.8–99.1)	77.7 (64.3–93.9)	94.8 (75.9–118.3)	85 (76.1–95)	85.2 (77.8–93.3)
Addis Ababa	76.8 (57.1–103.3)	55.8 (37.2–83.8)	60.6 (36.5–100.5)	63.4 (49.8–80.9)	66 (55.1–79.1)
Bulawayo	25.5 (7.1–91.3)	30.6 (13.9–67.6)	50 (30.7–81.4)	40.4 (26.6–61.5)	34.1 (22.7–51.1)
Cotonou	78 (54.7–111.2)	63.2 (40.1–99.7)	110.7 (86.7–141.5)	80.4 (63.8–101.2)	83.2 (70.9–97.7)
Eastern Cape	63.7 (50.2–80.8)	68.7 (56.7–83.2)	68.5 (57–82.4)	67.4 (59.9–75.7)	66.5 (60.1–73.6)
Eldoret	51.3 (19.3–136.7)	93.1 (74.9–115.8)	102.4 (102.4–102.4)	99.7 (85.1–116.8)	78.3 (62.3–98.4)
Harare (black)	67.1 (51.1–88.2)	74 (63.6–86.1)	67.2 (53.9–83.7)	70.8 (63.1–79.6)	69.1 (61.9–77.3)
Harare (white)	100.7 (100.7–100.7)	70.7 (50.3–99.3)	79.6 (60.2–105.1)	78.3 (64.6–95)	85.9 (78.5–94)
Kampala	75.6 (61.5–93)	65.7 (51.4–84)	69.2 (52–92.1)	69.7 (60.4–80.5)	70.9 (63.2–79.4)
Mauritius	82.7 (73.9–92.6)	85.7 (78.6–93.5)	82.6 (74.9–91.2)	84.3 (79.6–89.3)	83.6 (79.5–87.8)
Nairobi	91 (82.5–100.5)	76.7 (62.7–93.9)	91.1 (76.7–108.2)	86.8 (79.5–94.9)	86.9 (81–93.2)
Namibia	88.6 (73.2–107.3)	92 (74–114.3)	83.4 (57.5–121)	88.7 (76.7–102.6)	88.1 (78.2–99.2)
Seychelles	87.4 (75.8–100.9)	88 (77.7–99.6)	83.9 (72–97.9)	86.6 (79.7–94.1)	86.6 (80.9–92.6)
Total	79.5 (74.9–84.3)	75.9 (71.8–80.3)	79.2 (74.5–84.2)	78 (75.4–80.7)	78.4 (76.2–80.6)

Registry	Year 3 RS				Year 3 ASRS
	< 65	65–74	> = 75	All ages	
Abidjan	36.8 (21.6–62.5)	48.7 (32.3–73.4)	83.8 (48.7–144.3)	51.5 (38.6–68.8)	53.9 (41.9–69.3)
Addis Ababa	47 (24.3–90.9)	33.9 (17.2–66.7)	28.1 (9.5–83.4)	35 (21.1–58.1)	37.7 (26–54.6)
Bulawayo	–	33.6 (15.2–74.3)	–	15.4 (5.8–41.1)	–
Cotonou	41.1 (19.7–85.7)	–	72 (28.2–183.9)	37.2 (18.6–74.3)	–
Eastern Cape	45.8 (30.5–68.8)	53.9 (39.1–74.1)	53.8 (38.4–75.3)	51.5 (41.8–63.4)	50.5 (42.5–59.9)
Eldoret	-	82 (57.5–116.9)	47.6 (17.5–129.9)	60.1 (37.2–96.9)	-
Harare (black)	59.3 (42.8–82.2)	46.1 (34.5–61.5)	78.6 (61.3–100.8)	59.9 (50.5–70.9)	61.1 (53–70.4)
Harare (white)	81.2 (49.7–132.5)	70.2 (46.9–105.1)	85.9 (60.8–121.4)	79.7 (62.2–102.1)	79.4 (64.3–98)
Kampala	60 (43.9–82.1)	48.5 (33.1–71.2)	48.1 (28.8–80.6)	52.7 (41.8–66.4)	53.2 (44.3–64)
Mauritius	68.9 (58.1–81.7)	68.3 (58.8–79.4)	73 (61.5–86.7)	71.1 (64.6–78.4)	69.9 (64.6–75.7)
Nairobi	77.1 (63.6–93.6)	64.3 (47.4–87.1)	117.6 (99–139.6)	82 (71.5–94)	85.1 (77.1–94)
Namibia	85.4 (66.4–109.9)	84 (58.9–119.8)	55.7 (24.5–126.4)	81.7 (65–102.8)	76.4 (62.9–92.8)
Seychelles	70.9 (54.8–91.8)	67.1 (52.5–85.9)	73.4 (55.7–96.8)	70.7 (60.7–82.5)	70.6 (62.3–79.9)
Total	61.8 (56–68.2)	57.7 (52.5–63.5)	70.2 (63.2–77.9)	62.9 (59.4–66.7)	63.1 (60.1–66.1)

Registry	Year 5 RS				Year 5 ASRS
	< 65	65–74	> = 75	All ages	
Abidjan	–	–	76.3 (31.3–186.2)	55 (38.9–77.7)	–
Eastern Cape	41.6 (24.7–70)	50.9 (34.1–76)	48.1 (30.7–75.3)	48.2 (36.6–63.4)	46.2 (37–57.6)
Eldoret	–	68.1 (36.6–126.9)	47.6 (17.5–129.9)	75.6 (41.3–138.6)	–
Harare (black)	49.3 (31.7–76.8)	38.9 (27–56)	84.3 (59.8–118.8)	54.3 (43.5–67.9)	56.4 (46.7–68.2)
Harare (white)	81.2 (49.7–132.5)	52.4 (27.4–100.5)	90.6 (59.4–138.3)	76.8 (55.6–106.0)	75.6 (59.4–96.2)
Kampala	58.3 (41.5–81.8)	39.8 (24.3–65.3)	52.4 (27.4–100.1)	49.3 (37.5–64.8)	51.2 (40.9–64.1)
Mauritius	63.5 (51.7–77.9)	68.6 (57.3–82.2)	75 (60–93.9)	70.0 (62.1–79.0)	68.3 (62–75.3)
Nairobi	65.7 (49.8–86.7)	60.7 (39.8–92.8)	152.6 (121.4–191.8)	80.7 (66.9–97.5)	89.5 (78.2–102.3)
Namibia	90.8 (70.6–116.8)	88.9 (54.9–144.1)	55.7 (24.5–126.4)	88.4 (68.4–114.3)	80.1 (64.9–98.7)
Seychelles	55 (37.6–80.3)	54.6 (37.1–80.4)	71.4 (49.1–103.8)	59.4 (47.4–74.4)	59.6 (49.8–71.4)
Total	56.1 (49.8–63.2)	51.8 (45.7–58.7)	74.9 (65.3–85.9)	60 (55.7–64.6)	60.3 (56.7–64.1)

*ASRS* age-standardized relative survival, *RS* relative survival

**Table 6:** Relative survival (RS) by stage and registry

Registry	*n*	No. with known stage	No. Stage IV (%)	1 Year RS (95% CI)											
				Stage I + II	Stage III	Stage IV	Stage unknown	Stage I + II	Stage III	Stage IV	Stage unknown	Stage I + II	Stage III	Stage IV	Stage unknown
Abidjan	127	46	22 (48)	74 (38–142)	89 (70–112)	63 (44–91)	88 (79–99)	74 (38–142)	62 (33–116)	31 (14–70)	53 (37–75)	–	81 (43–151)	–	–
Addis Ababa	45	4	4 (100)	–	–	100 (100–100)	59 (45–78)	–	–	–	35 (22–58)	–	–	–	–
Bulawayo	50	19	10 (53)	–	54 (29–101)	32 (13–80)	38 (20–69)	–	–	16 (4–63)	–	–	–	–	–
Cotonou	43	31	13 (42)	110 (110–110)	105 (86–127)	35 (15–84)	97 (79–118)	–	41 (15–114)	–	87 (51–147)	–	–	–	–
Eldoret	23	12	6 (50)	100 (100–100)	100 (100–100)	70 (42–117)	113 (113–113)	62 (23–164)	100 (61–163)	58 (29–118)	27 (7–103)	–	128 (57–289)	58 (22–152)	27 (7–103)
Harare (black)	148	37	7 (19)	100 (100–100)	74 (57–96)	61 (34–109)	70 (61–80)	65 (29–147)	38 (21–69)	–	67 (57–80)	48 (13–172)	8 (2–35)	–	68 (55–84)
Harare (white)	41	10	3 (30)	100 (100–100)	73 (38–141)	38 (12–124)	80 (65–99)	100 (100–100)	73 (38–141)	38 (12–124)	78 (58–105)	100 (100–100)	46 (14–149)	–	81 (57–116)
Kampala	114	31	17 (55)	41 (20–83)	100 (100–100)	53 (33–85)	77 (67–89)	36 (15–84)	73 (27–194)	28 (11–67)	61 (49–78)	–	–	24 (8–72)	61 (47–79)
Nairobi	134	50	25 (50)	103 (103–103)	74 (48–114)	89 (75–106)	84 (74–95)	109 (109–109)	74 (48–114)	57 (36–90)	84 (71–99)	125 (125–125)	65 (34–124)	29 (11–78)	89 (73–109)
Namibia	35	29	15 (52)	100 (100–100)	101 (101–101)	81 (61–107)	70 (42–117)	114 (114–114)	101 (101–101)	55 (32–95)	61 (30–124)	128 (128–128)	127 (127–127)	46 (22–95)	61 (30–124)
Seychelles	119	87	39 (45)	94 (84–105)	95 (84–107)	80 (68–95)	79 (65–97)	97 (80–118)	78 (57–105)	45 (31–67)	75 (57–99)	95 (72–125)	83 (59–117)	27 (14–53)	48 (29–80)
Total	879	356	161 (45)	91 (83–100)	87 (78–96)	70 (62–78)	76 (72–81)	89 (78–103)	61 (49–76)	38 (30–49)	64 (58–71)	94 (79–113)	56 (42–76)	24 (15–37)	63 (56–71)

## Code availability

The custom code used around the packages described in the methods section is available from the corresponding author upon reasonable request.
